# An Analysis of Intrinsic Protein Disorder in Antimicrobial Peptides

**DOI:** 10.1007/s10930-025-10253-0

**Published:** 2025-02-20

**Authors:** Michael Antonietti, Colin K. Kim, Sydney Granack, Nedym Hadzijahic, David J. Taylor Gonzalez, William R. Herskowitz, Vladimir N. Uversky, Mak B. Djulbegovic

**Affiliations:** 1 Bascom Palmer Eye Institute, University of Miami, Miami, FL USA; 2 Department of Molecular Medicine and USF Health Byrd Alzheimer’s Research Institute, Morsani College of Medicine, University of South Florida, Tampa, FL USA; 3 University of Miami, Miami, FL USA; 4 Wills Eye Hospital, Thomas Jefferson University, Philadelphia, PA USA; 5 Hamilton Eye Institute, University of Tennessee Health and Science Center, Memphis, United States

**Keywords:** Antimicrobial peptides, intrinsic protein disorder, liquid-liquid phase separation, proteomics

## Abstract

Antibiotic resistance, driven by the rise of pathogens like VRE and MRSA, poses a global health threat, prompting the exploration of antimicrobial peptides (AMPs) as alternatives to traditional antibiotics. AMPs, known for their broad-spectrum activity and structural flexibility, share characteristics with intrinsically disordered proteins, which lack a rigid structure and play diverse roles in cellular processes. This study aims to quantify the intrinsic disorder and liquid–liquid phase separation (LLPS) propensity in AMPs, advancing our understanding of their antimicrobial mechanisms and potential therapeutic applications. To investigate the propensity for intrinsic disorder and LLPS in AMPs, we compared the AMPs to the human proteome. The AMP sequences were retrieved from the AMP database (APD3), while the human proteome was obtained from the UniProt database. We analyzed amino acid composition using the Composition Profiler tool and assessed intrinsic disorder using various predictors, including PONDR® and IUPred, through the Rapid Intrinsic Disorder Analysis Online (RIDAO) platform. For LLPS propensity, we employed FuzDrop, and FuzPred was used to predict context-dependent binding behaviors. Statistical analyses, such as ANOVA and χ^2^ tests, were performed to determine the significance of observed differences between the two groups. We analyzed over 3000 AMPs and 20,000 human proteins to investigate differences in amino acid composition, intrinsic disorder, and LLPS potential. Composition analysis revealed distinct differences in amino acid abundance, with AMPs showing an enrichment in both order-promoting and disorder-promoting amino acids compared to the human proteome. Intrinsic disorder analysis, performed using a range of predictors, consistently demonstrated that AMPs exhibit higher levels of predicted disorder than human proteins, with significant differences confirmed by statistical tests. LLPS analysis, conducted using FuzDrop, showed that AMPs had a lower overall propensity for LLPS compared to human proteins, although specific subsets of AMPs exhibited high LLPS potential. Additionally, redox-dependent disorder predictions highlighted significant differences in how AMP and human proteins respond to oxidative conditions, further suggesting functional divergences between the two proteomes. CH-CDF plot analysis revealed that AMPs and human proteins occupy distinct structural categories, with AMPs showing a greater proportion of highly disordered proteins compared to the human proteome. These findings underscore key molecular differences between AMPs and human proteins, with implications for their antimicrobial activity and potential therapeutic applications. Our study reveals that AMPs possess a significantly higher degree of intrinsic disorder and specific subsets exhibit LLPS potential, distinguishing them from the human proteome. These molecular characteristics likely contribute to their antimicrobial function and adaptability, offering valuable insights for developing novel therapeutic strategies to combat antibiotic resistance.

## Introduction

As science continues to progress, our understanding of pathogens and their effect on living organisms advances as well. Specifically, the treatment of bacterial infections has drastically evolved in recent years. The use of traditional antibiotic medicines in common practice has caused drug resistance to become an issue on a global scale, leading to an evolving epidemic where “superbugs,” such as vancomycin-resistant Enterococcus (VRE) and methicillin-resistant Staphylococcus aureus (MRSA), are a serious threat to patients and present a burden to healthcare systems [1–3].

To potentially address this healthcare challenge, the field of antimicrobial peptides (AMPs) has evolved [4–7]. AMPs are small molecular peptides that play a crucial role in host innate immunity and have been the focus of current efforts to create treatments for antibiotic-resistant pathogens that differ from traditional antibiotics [5, 6, 8]. They come from synthetic or natural sources and contain a broad range of antimicrobial activity against parasites, fungi, and other microorganisms [5, 6, 8]. AMPs are recognized as a primordial aspect of the host immune system, found preserved across eukaryotes, and possess the ability to target pathogens faster within a host compared to immunoglobulins [5, 8, 9]. They are also often found in locations that are vulnerable to pathogens, such as the mucous membranes [5, 8].

AMPs are categorized based on their net charge and protein structure. The cationic charge of AMPs can disrupt cell membranes of pathogens, inhibiting their proper function [2, 5]. In addition, conformational changes, the peptide-lipid ratio, peptide length, net charge, hydrophobicity, and secondary structure all influence the interactions between AMPs and cell membranes of potential pathogens [5, 8]. AMPs also contain a strong targeting specificity for pathogens that may cause harm to an organism [2, 7]. These features of AMPs highlight their potential as therapeutic agents and also invite comparisons with other protein classes, such as intrinsically disordered proteins (IDPs).

Since the turn of the century, the molecular behavior of peptides has been brought into question. The perception that peptides are three-dimensional, relatively rigid structures that serve one main purpose has been challenged. As our understanding of protein structure has evolved, a growing body of research has emerged, focusing on proteins that defy the conventional rigid structure model known as intrinsically disordered proteins (IDPs) [10, 11]. IDPs lack a strict three-dimensional structure and possess a high degree of flexibility, existing as a conformational ensemble, allowing for a broad range of physiologic activity [10, 12]. Intrinsic disorder can be observed at a global scale with the lack of the overall three-dimensional structure of an entire protein and also at a local scale within the proteins, where regions lack of secondary structure like α-helices and β-sheets [13]. The existence of IDPs support the idea that proteins can perform necessary cellular functions without a stable, three-dimensional structure under physiological conditions [10, 14, 15]. Both ordered and intrinsically disordered regions (IDRs) can coexist within a single protein [10]. IDPs are seen in increasing prevalence with more complex organisms. While AMPs are found in both eukaryotes and prokaryotes, higher-order eukaryotes have an increased propensity for intrinsic disorder compared to their prokaryotic counterparts [16, 17]. This reflects the various complex needs for regulation in eukaryotic cells [16].

In addition, the awareness of liquid–liquid phase separation (LLPS) is vital to understanding the function and role of intrinsic protein disorder in humans, prokaryotes, and AMPS. LLPS occurs when, under specific pH and concentration conditions, a homogenous mixture separates into two distinct liquid phases without the involvement of a solid phase [18, 19]. Although the relationship between IDPs and LLPS is still being investigated, the fact that IDPs are particularly prone to undergoing this process is clear due to their lack of a rigid structure. During LLPS, proteins, for example IDPs, condense and form droplet-like structures, effectively isolating themselves from the rest of the cellular environment. This phase separation results in the creation of membrane-less compartments within the cell, which serve to compartmentalize biochemical reactions and regulate various cellular processes [20, 21]. The reversible nature of LLPS makes it a practical and dynamic mechanism in living organisms, allowing cells to rapidly respond to changing conditions.

Similar to IDPs and their propensity to undergo LLPS, most AMPs exist in unstructured states such as random coils or extended conformation when in an aqueous solution [22, 23]. When certain AMPs come into contact with a bacterial membrane, they can adapt an amphiphilic α-helix, compromising the stability of the membrane and eventually killing the pathogen [24]. The ability of both IDPs and AMPs to change structure to bind multiple partners allows for quick changes in function [8, 10]. Given the structural flexibility and functional versatility of both IDPs and AMPs, it is important to further investigate whether AMPs exhibit significant levels of intrinsic disorder and a propensity for LLPS. The overlapping characteristics between these protein classes suggest that intrinsic disorder might be an inherent feature of AMPs, contributing to their antimicrobial activity. By understanding the extent of intrinsic disorder and LLPS within AMPs, we can gain deeper insights into their mechanisms of action, potentially identifying new pathways for addressing antibiotic resistance. This study, therefore, seeks to systematically characterize AMPs in the context of intrinsic disorder and LLPS, advancing our understanding of their molecular interactions and functional roles.

In our analysis, we aim to quantify and categorize the level of intrinsic protein disorder and the propensity for LLPS within a large database of AMPs as compared to the human proteome. As there is a key overlap between IDPs and AMPs, we posit that there is a distinct difference in intrinsic disorder between the AMPs and the human proteome. For our analysis of AMPs, we utilized the Data Repository of Antimicrobial Peptides, which has reported and identified nearly 4,000 different AMPs [25–27]. The human proteome utilized in this analysis was derived from the Universal Protein (UniProt) database [28, 29]. The purpose of this study is to provide the most comprehensive analysis to date of AMPs as they relate to their propensity of intrinsic disorder and LLPS, using a similar computational methodology as previously described by our group [30–32] By quantifying the levels of intrinsic protein disorder and their tendency to undergo LLPS in the AMPs as it compares to the human proteome, we may be able to further understand the role of intrinsic protein disorder in AMPs, shedding new light on their interactions. We hope this analysis will enhance our understanding of the relationship between intrinsic protein disorder and AMPs.

## Methods

### Protein Identification

**Fig. 1:**
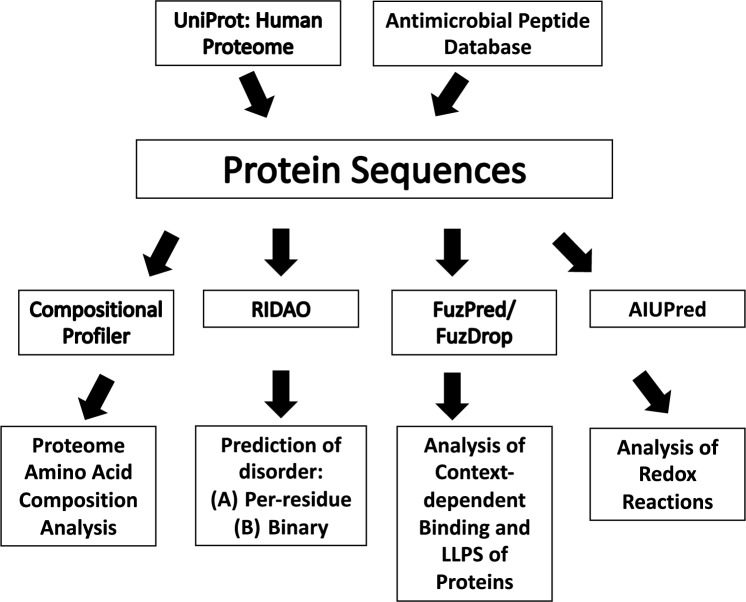
Workflow diagram summarizing methodological steps to analyze intrinsic disorder and liquid–liquid phase separation of human proteome and antimicrobial peptides (AMPs). *RIDAO* rapid intrinsic disorder analysis online, *LLPS* liquid–liquid phase separation

Our study aim is to compare the propensity of intrinsic protein disorder and LLPS of two distinct groups: the human proteome and antimicrobial peptides. The human proteome and AMPs required different retrieval procedures. The AMPs were collected as a set FASTA sequences from the AMP database (APD3) at the University of Nebraska Medical Center (available at https://aps.unmc.edu/downloads, accessed on 3/25/24) [25–27]. In addition, the human proteome sequences were retrieved from the well-known Universal Protein database (UniProt; available at https://www.uniprot.org/, accessed on 3/25/24) [29]. The UniProt database was filtered by “Swiss-Prot” (i.e., reviewed entries) and by popular organism, “Human” (Fig. 1).

We note that certain tools used in this study impose their own input criteria (e.g., minimum or maximum sequence lengths), which can lead to a small number of sequences being automatically excluded from some analyses. Consequently, the final sequence counts reported in individual method sections may differ slightly from the total number of sequences initially identified. This discrepancy is a recognized artifact of using multiple specialized tools, each with its own constraints, and does not compromise the overall representativeness of our results.

### Amino Acid Composition Analysis

The Composition Profiler (CP) tool (available at http://www.cprofiler.org/, accessed on 3/26/24) was used to analyze the AMP and the human proteomes [33]. The tool enables visualization and measurement of the frequency of each amino acid within these proteomes. AMPs and human proteomes were query sets against a standard reference set called 'Protein Data Bank Select 25' as defined by the CP [34]. Additionally, comparison profiles were created using the DisProt database, which catalogs intrinsically disordered proteins, and the SwissProt database, recognized for reflecting natural amino acid distributions [35, 36] Amino acids were assessed for their role in either promoting order or disorder as it relates to protein structure. Amino acids that were enriched resulted in positive values, whereas those that were depleted resulted in negative values. The ten amino acids that are order-promoting residues are C, W, I, Y, F, L, H, V, N, and M, while those that are disorder-promoting residues are R, T, D, G, A, K, Q, S, E, and P. The degree of enrichment or depletion of an amino acid was determined by the formula (Cx–Corder)/Corder, where Cx is the concentration of the amino acid in the protein being studied, and Corder is its concentration in the PDB Select 25 reference set.

### Analysis of Intrinsic Protein Disorder and Liquid–Liquid Phase Seperation

#### Prediction of Disorder Using Commonly Used Predictors

In the next stage of our analysis, we used the Rapid Intrinsic Disorder Analysis Online (RIDAO) tool (available at https://RIDAO.app, accessed on 3/28/24) to examine the intrinsic disorder at the residue level of amino acids [37]. We calculated the Average Disorder Scores (ADS) and Percentages of Predicted Disordered Residues (PPDR) for each protein using a suite of tools that are part of the RIDAO platform. The tools included four versions of the Predictor of Natural Disordered Regions (PONDR®): VSL2, VL3, VLXT, and FIT, as well as the IUPred tool in both its Short and Long versions. The ADS measures a protein's overall disorder propensity, and the PPDR quantifies the proportion of amino acids likely disordered, with scores above 0.5 indicating significant disorder. While ADS provides a global average, PPDR assesses the extent of disorder across amino acids. Therefore, evaluating ADS independently is crucial for a comprehensive protein analysis. A protein is tagged as highly ordered if its ADS is below 0.15, moderately disordered if the ADS is between 0.15 and 0.5, and highly disordered if the ADS is 0.5 or above.

We used Analysis of Variance (ANOVA) tests to compare the ADS and PPDR scores between the two groups in our study, the AMPs and the human proteome. With ANOVA analysis, we determined if the differences in disorder levels observed between the groups were statistically significant or if they could be attributed to random variation.

#### Average Disorder vs Percent of Predicted Disorder Residues Analysis

As we proceeded with our per-residue disorder analysis, we applied the Predictor of Natural Disordered Regions Version 2 (PONDR® VSL2) from the RIDAO output [37–39]. This tool is tailored to assess the degree of disorder at the amino acid residue level within proteins and its accuracy has been affirmed through the Critical Assessment of Protein Intrinsic Disorder [40]. Utilizing the data from PONDR® VSL2, we set specific thresholds to classify each protein by its degree of disorder, adhering to criteria established in our earlier works [31, 41, 42]. We designated proteins as highly ordered if their Percentage of Predicted Disordered Residues (PPDR) was below 10% or their Average Disorder Score (ADS) was under 0.15. Proteins were considered moderately disordered with a PPDR of 10% to less than 30%, or an ADS between 0.15 and just below 0.5. A designation of highly disordered was given to proteins with a PPDR of 30% or higher and an ADS of 0.5 or above [31, 43, 44]. This classification scheme enables a nuanced investigation into the structural organization of proteins. Again, it is important to remember the key differences between ADS and PPDR, refer to Methods Sect. 2.3.1 for more information.

To investigate significant differences in protein disorder between the AMPs and the human proteome, we employed χ^2^ tests. These tests allowed us to determine if the distribution of proteins across different disorder categories—specifically “Ordered” versus “Disordered”—varied significantly between the two groups. By comparing the populations of proteins within each category, we could assess whether the observed differences in disorder levels were statistically significant, rather than occurring by chance.

To ensure the assumptions of the χ^2^ test were met, particularly that each expected count exceeded 5, we grouped the “Highly Ordered” and “Moderately Ordered or Mildly Flexible” categories into a single “Ordered” category. This grouping was necessary to avoid issues such as division by zero and to maintain the statistical validity of the analysis. By making this adjustment, we ensured the χ^2^ test could be performed reliably, providing accurate and interpretable results regarding the distribution differences between Human and AMPs.

#### Analysis of Context-dependent Binding Behavior of Proteins (FuzPred)

Next, we assessed the functional differences between the AMP and human proteomes. This was achieved using FuzPred, a tool that predicts the context-dependent binding behavior of proteins (available at https://fuzpred.bio.unipd.it/predictor, accessed on 6/15/24). The FuzPred tool can estimate the context-dependent binding behavior of proteins [45, 46]. FuzPred determines a series of output metrics for each protein residue, which provides further understanding of their potential function and interaction behavior as measured by the following measures: (1) Probability of Disorder-to-Order (pDO): This metric anticipates the propensity of a protein conforming to a stable, ordered structure once bound to a cellular partner. The higher the pDO value for a protein the higher its likelihood to transition from a disordered to an ordered state upon binding. (2) Probability of Disorder-to-Disorder (pDD): pDD predicts the chance that a residue will remain disordered and maintain conformationally flexibility when it binds to another cellular component. An increase in the pDD value indicates a greater possibility of the protein remaining disordered during binding. (3) Multiplicity of Binding Modes (MBM): MBM is a metric which shows the variety of a protein’s binding and interactions across various partners and environmental conditions. A high MBM value suggests that a residue’s interactions are likely context-dependent and will exhibit a high likelihood for aggregation if the protein is disordered.

FuzPred analytics of pDO, pDD, and MBM, can be compounded to allow for classification of each amino acid in the protein under examination. There are four categorizations that are outputted by FuzPred. (1) The structured Binding classification are proteins associated with regions that consistently adopt a well-defined structure upon binding with all their interaction partners. These proteins have a low pDD, high pDO, and low MBM which indicates a level of stability across cellular conditions. (2) The LLPS-promoting classification examines amino acids that prefer forming disordered complexes and often participate in LLPS. These proteins have high pDD, low pDO, and low MDM values which means they favor disorder across various conditions. (3) Disordered Assemblies look at proteins that form disordered structures but can switch to ordered versions under specific partners or conditions. These proteins have high pDD, low pDO, and high MBM values. They are referred to as ‘Conditional Assemblers’ due to this duality and are often found in aggregation hot spots. Finally, (4) Disorder-to-Order binders identify proteins called ‘Polymorphic Binders’ which often are associated with amyloid cores. These amino acids have a low chance of remaining disordered once binding is complete (low pDD/high pDO) but have a high MBM, indicating many disorder-to-order structural shifts that can form polymorphic proteins.

#### Analysis of Protein LLPS Probability (FuzDrop)

The functional differences between the human proteome and AMPs can also be assessed through the utilization of the web server FuzDrop (available at https://fuzdrop.bio.unipd.it/predictor, accessed on 6/15/24). This tool analyzes amino acid inputs for their various disorder and order regions. FuzDrop predicts the probability of proteins to undergo LLPS by sequence-based identification of droplet-promoting regions and aggregation-promoting regions within droplets [47]. Droplet-promoting regions are associated with IDPs while aggregation-promoting regions are common in more ordered β-sheet-rich aggregates. Like FuzPred, FuzDrop also does not specify binding partners for each prediction.

There are a series of output analytics which can be manipulated to further assess the likelihood of LLPS occurring within an amino acid sequence. Residue-based droplet-promoting probabilities (pDP) refer to how likely a certain protein region is to form droplet residues or biomolecular condensates. pDP assesses the propensity of a residue to undergo LLPS, forming into a droplet structure. Droplets formed through LLPS often involve proteins with high intrinsic disorder, which contribute to dynamic interactions within transient, membrane-less compartments essential for various cellular processes [18, 47]. This function allows for compartmentalization of reactions in a specific protein-partner interaction. LLPS probability, specifically, pDP, can signal a deeper understanding of the disorder and order within the specified region. Droplet-promoting regions (DPRs) and aggregation hot spots are the opposing identification regions based on pDP. Finally, there are a series of sequence features provided in the FuzDrop output. Fuzzy regions have structural flexibility and binding variation which allows them to be a contributing component in IDPs and LLPS [45]. Post-translational modifications (PTMs) and protein families (Pfam) are additional identifying sequence features that are shown by FuzDrop. Each of these regions is associated with determining the context-dependence of the examined protein. FuzDrop creates an AlphaFold predicted protein structure identifying blue droplet-promoting regions and orange aggregation hot spots.

#### Analysis of Redox Reactions with AIUPred

To further our analysis of the functional implication of intrinsic disorder within the AMP and human proteome, we utilized a valuable computational tool called AIUPred (available at https://iupred.elte.hu/, accessed on 6/15/24). AIUPred is a predictive tool that incorporates a transformer neural network within its framework to enhance the understanding of protein residues and their cellular properties and corresponding functions. This computational mechanism analyzes the interactions between various proteins and their amino acids simultaneously, allowing AIUPred to draw accurate conclusions about both short-range and long-range interconnectivity of amino acid sequences–providing insights into the interconnectivity of amino acid sequences. By inputting these sequences, AIUPred estimates positional energies and translates them into predictions about intrinsically disordered regions (IDRs). The process begins with an input of amino acid sequences, which are then processed through the transformer model to estimate positional energies. Following this, AIUPred translates these energy estimates into output metrics and predictions regarding protein disorder, improving the accuracy of identifying intrinsically disordered regions in proteins.

AIUPred identifies IDRs by evaluating each amino acid’s potential for disorder, considering factors like sequence and cellular conditions. The tool offers multiple functionalities, including predictions of protein disorder (AIUPred), binding regions (ANCHOR 2), and redox-dependent disorder. Our study focuses on the redox-dependent disorder prediction feature of AIUPred, which examines how the disorder propensity of amino acids shifts under different redox states in the protein’s environment. This is crucial because redox conditions significantly influence protein structure and function. By analyzing redox-dependent disorder, we aim to gain insights into how these proteins might behave under different oxidative conditions, which may be crucial for understanding their antimicrobial mechanisms and potential applications.

### CH-CDF Plot Analysis

In our expanded analysis, we incorporated two binary predictors of disorder: the charge-hydropathy (CH) analysis and the cumulative distribution function (CDF). These tools were applied to assess the overall intrinsic disorder within the entire proteins of both groups. This method differs from our prior residue-level evaluation by providing a binary output, indicating whether a protein is disordered or ordered, unstructured or structured. The CH predictor calculates disorder by considering the net charge and hydropathy of the proteins, where disordered proteins usually show a higher net charge and a lower hydropathy. On the other hand, the CDF plots the proportion of disordered amino acids across the protein’s length. By merging CH and CDF data, we generated CH-CDF plots to visually compare every protein within the antimicrobial peptide and human proteome groups, thus facilitating a broad comparative analysis.

The CH-CDF plot is a method that allows us to categorize proteins as either structured or unstructured by their location on the chart, which uses a Cartesian coordinate system for easy qualitative analysis. This system aids in the detailed evaluation of each protein’s structural tendencies on a two-dimensional scale. Proteins in Quadrant 1 (Q1, bottom right) are deemed likely to be structured, as indicated by their negative CH scores and positive CDF scores. Proteins in Quadrant 2 (Q2, bottom left) might be classified as molten globules or hybrids; they have negative scores for both CH and CDF, which implies a level of compactness without significant structure or disorder. Proteins in Quadrant 3 (Q3, top left) are considered highly disordered, with positive CH scores and negative CDF values. Lastly, proteins in Quadrant 4 (Q4, top right) show conflicting indicators, being tagged as disordered in the CH assessment but ordered in the CDF evaluation [44, 48].

### Length-Adjusted Comparison of Human and AMPs

To address potential length-related biases, a subset of human proteins with sequence lengths comparable to AMPs was identified and analyzed. Human protein sequences were retrieved from the UniProt database and filtered to include only those within the range of sequence lengths observed for AMPs. This length-matched subset was then analyzed alongside the AMP dataset for intrinsic disorder and LLPS propensities.

Intrinsic disorder was assessed using the RIDAO tool, which provided ADS and PPDR each protein. LLPS propensity was evaluated using FuzDrop, identifying droplet-promoting regions indicative of phase separation. Additionally, the binding transition probabilities (pDO and pDD) for context-dependent binding behaviors were analyzed using FuzPred.

To account for non-normal data distributions and unequal variances between AMPs and the length-matched subset of human proteins, statistical comparisons of LLPS metrics (pLLPS, pDO, and pDD) were performed using the Mann–Whitney U test. This non-parametric method ensured robust comparisons and identified significant differences while accommodating the dataset’s distributional characteristics.

## Results

### Protein Identification

We analyzed 3172 AMPs and 20,435 human proteins in FASTA format using a range of bioinformatics tools. The sequence counts in some parts of our analysis were lower than expected because certain tools excluded sequences that were too short for processing. We organized our analysis into two distinct groups: the AMPs and the human proteome, terms that were consistently used throughout all subsequent analyses.

### Amino Acid Composition Analysis

**Fig. 2:**
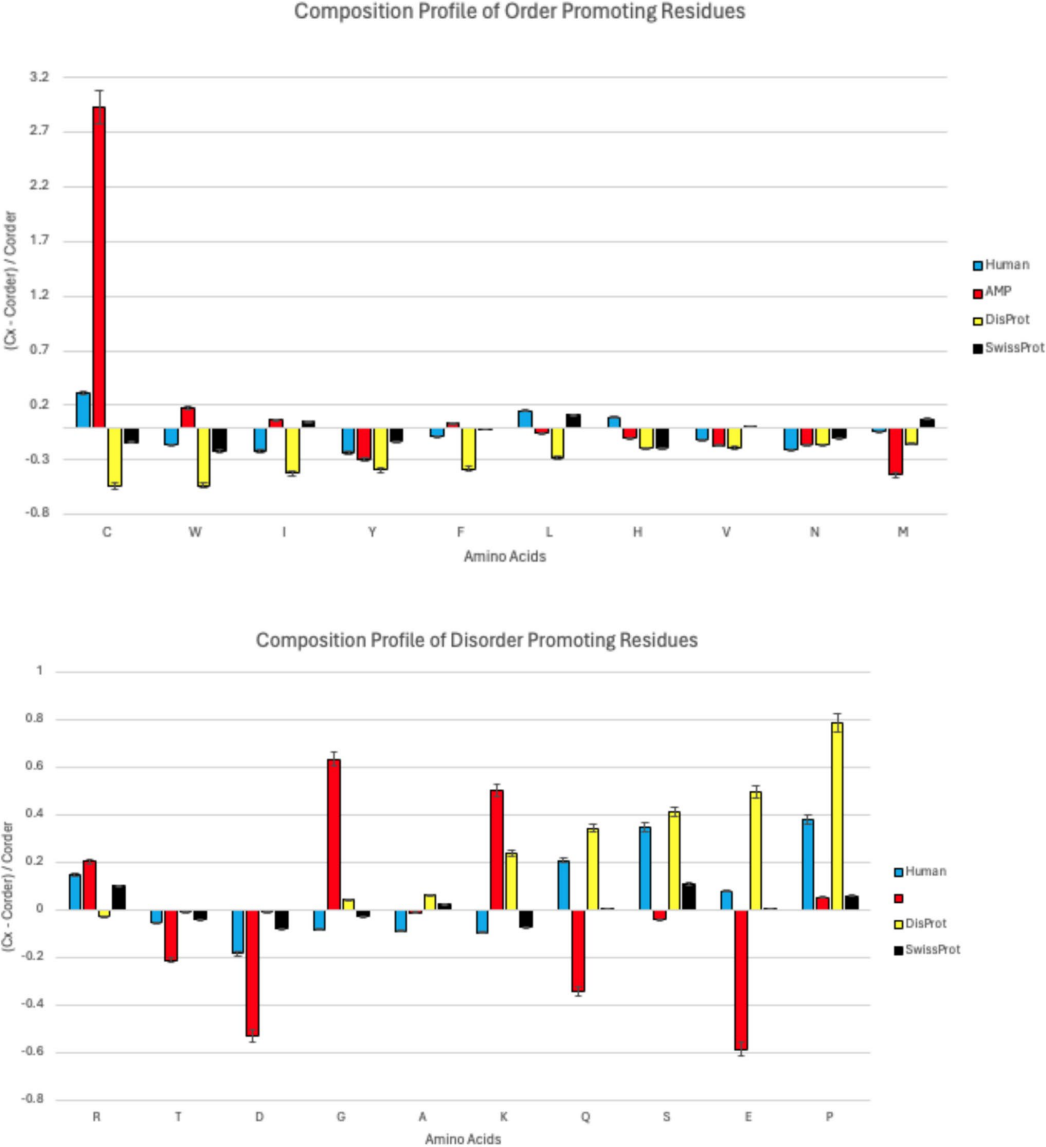
Amino acid composition profile of 1373 AMP (red bar) and 20,352 human proteins (blue bar). The fractional difference is calculated as (C_x_ − C_order_)/C_order_, where C_x_ is the content of a given amino acid in the query set (1,373 AMPs, 20,352 human proteins or proteins from the SwissProt database), and C_order_ is the content of a given amino acid in the background set (Protein Databank Select 25). The amino acid residues are ranked from most order-promoting residue to most disorder-promoting residue. Positive values indicate enrichment and negative values indicate depletion of a particular amino acid. The composition profile generated for experimentally validated disordered proteins from the DisProt database (black bars) and the distribution of amino acids in nature from the SwissProt database (yellow bars) is shown for comparison. The error bars correspond to standard deviations over 10,000 bootstrap iterations. In the antimicrobial peptides (AMPs), among the ten amino acids known to promote order four (C, W, I, and F) were found abundantly. In contrast, the human proteome exhibited abundance in only three of these order-promoting amino acids (C, L, and H). Within the AMPs four disorder-favoring amino acids (R, G, K, P) were prevalent. Meanwhile, proteins in the human proteome displayed abundance in five amino acids (R, Q, S, E, and P) typically associated with promoting disorder

The amino acid profiles of the AMPs and the human proteome were quantified. Their amino acid profiles were juxtaposed to experimentally verified protein composition profiles from the DisProt database and the natural amino acid distribution found in the SwissProt database (Fig. 2). The amino acids were organized according to their tendency to induce structural order or disorder within the proteins, with positive values indicating an abundance of an amino acid, and negative values indicating a scarcity. In the AMPs, of the ten amino acids known to encourage order (C, W, I, Y, F, L, H, V, N, and M), four (C, W, I, and F) were found to be in abundance. In contrast, the human proteome showed an abundance in only three of the order-promoting amino acids (C, L, and H). Within the human proteome, 5 of 10 disorder promoting amino acids residues (R, Q, S, E, and P) were enriched. For the antimicrobial peptides, four disorder-promoting amino acids (R, G, K, P) were enriched.

### Analysis of Intrinsic Protein Disorder and Liquid–Liquid Phase Separation

#### Prediction of Disorder Using Commonly Used Predictors

The subsequent objective of our study was to assess and compare the patterns at an amino acid level between the two groups. These sets were distinguished by proteins unique to and shared between AMPs and those from the human proteome. For this analysis, we considered the protein comparison on a one-to-one basis. Importantly, when we used RIDAO to analyze the AMPs, the tool filtered the FASTA sequences based on length. The starting database contained 3172 proteins, but only 1373 of those were able to be analyzed by RIDAO. The remaining 1799 AMPs were filtered out by RIDAO because their sequences were too short.

We utilized the RIDAO tool to obtain disorder measurements for further comparative analysis of disorder profiles, employing two distinct scoring metrics: the percentages of predicted disordered residues (PPDR) and Average Disorder Scores (ADS) for each type of disorder predictor (Table 1). See Methods Sect. 2.3.1 for more details on these metrics. This analysis involved predictions of intrinsic disorder for both the AMPs and human proteome, using a variety of per-residue amino acid predictors. Using the PONDR® VLXT predictor, PPDR for the AMPs and human proteome was 29.66% and 26.46%, respectively, while the ADS was 0.33 for the AMPs and 0.3 for the human proteome. The ANOVA test yielded F-statistics of 5.32 and 7.21 (P-values of < 0.01 for both PPDR and ADS) for PONDR® VLXT, indicating statistically significant differences.

**Table 1:** Table showing the average disorder scores (ADS) and percentages of predicted disordered residues (PPDR) of each of the six disorder predictors and the statistical differences in these values among the antimicrobial peptides, and human proteome

	Antimicrobial peptides (%)	Human proteome (%)	ANOVA F-statistic	ANOVA P-value
PPDR-VLXT	29.66	26.46	5.32	< 0.01*
ADS-VLXT	0.33	0.3	7.21	< 0.01*
PPDR-VSL2B	32.15	28.51	24.09	< 0.0001*
ADS-VSL2B	0.42	0.39	25.59	< 0.0001*
PPDR-VL3	24.54	19.6	20.81	< 0.0001*
ADS-VL3	0.34	0.31	23.63	< 0.0001*
PPDR-IUP-Short	13.85	11.45	7.16	< 0.01*
ADS-IUP-Short	0.25	0.24	13.22	< 0.0001*
PPDR-IUP-Long	13.77	11.25	7.69	< 0.01*
ADS-IUP-Long	0.27	0.26	14.26	< 0.0001*
PPDR-PFIT	20.46	16.25	10.11	< 0.0001*
ADS-PFIT	0.3	0.27	13.29	< 0.0001*
PPDR-MDP	17.87	13.81	9.18	< 0.001*
ADS-MDP	0.3	0.27	17.11	< 0.0001*

*PPDR* percentages of predicted disordered residues, *ADS* average disorder scores, *MDP* mean disorder profile

*Statistically significant, p-value < 0.05

The PONDR® VSL2 and VL3 models indicate significant differences in protein disorder between AMPs and the human proteome, as shown by PPDR values (32.15 vs. 28.51% for VSL2 and 24.54 vs. 19.60% for VL3) and ADS values (0.42 vs. 0.39 for VSL2 and 0.34 vs. 0.31 for VL3), with both showing statistical significance (P < 0.0001).

IUPred Short (IUP_S) and IUPred Long (IUP_L) also show significant differences in PPDR and ADS, highlighting the varied levels of disorder within AMPs compared to the human proteome. Specifically, IUP_S ranges from 13.85 to 11.45% for PPDR, with corresponding ADS values from 0.25 to 0.24, while IUP_L ranges from 13.77 to 11.25% for PPDR, with ADS from 0.27 to 0.26, both demonstrating statistical significance (P < 0.01 for PPDR and P < 0.0001 for ADS in both models).

The PONDR® FIT and MDP models further support these findings, with PPDR values ranging from 20.46% to 16.25% and ADS from 0.3 to 0.27 for FIT, and 17.87 to 13.81% for PPDR and 0.3 to 0.27 for ADS in MDP, all indicating significant differences (P < 0.0001 for both metrics in both models).

#### Average Disorder vs Percent of Predicted Disorder Residues Analysis

**Fig. 3:**
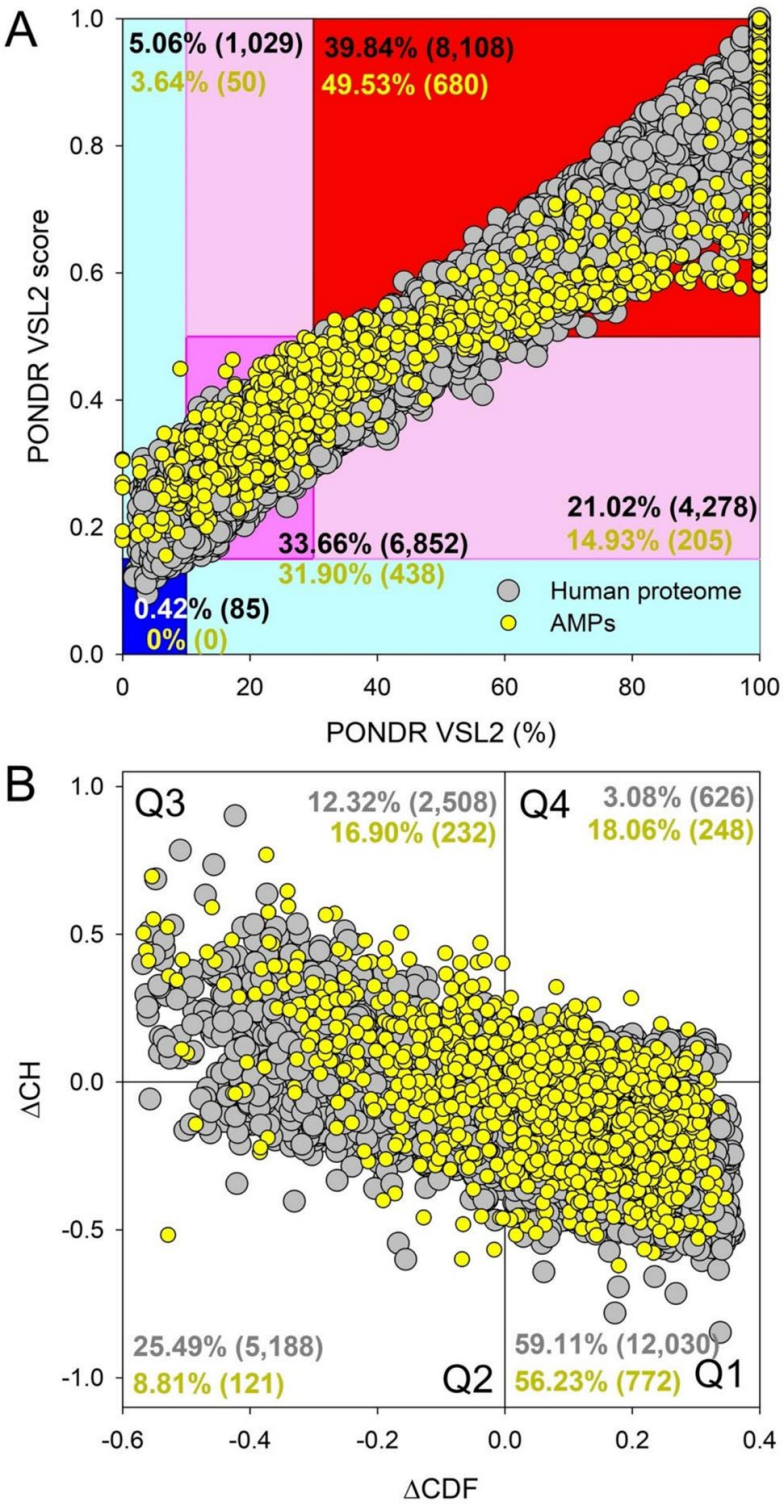
Multidimensional protein disorder analysis in AMPs and human proteins. **A** PONDR® VSL2 Score vs. VSL2 PONDR®(%) analysis showing AMP and human proteins with general agreement between each group. PONDR® VSL2 (%, x-axis, **A**) is a percent of predicted disordered residues (PPDR) from 0 to 100%, i.e., residues with disorder scores above 0.5. Of the proteins analyzed, 8108 (39.84%) human proteins, and 680 (49.53%) AMPs are predicted to be highly disordered. The PONDR® VSL2 average disorder score (ADS) (y-axis, **A**) is a protein’s average disorder score from 0 to 1 that provides a global average measure of disorder across a protein sequence. Color blocks indicate regions in which proteins are mostly ordered (blue and light blue), moderately disordered (pink and light pink), or mostly disordered (red). If the two parameters agree, the corresponding part of the background is dark (blue or pink), whereas light blue and light pink reflect areas where the predictors disagree with each other. The boundaries of the colored regions represent arbitrary and accepted cutoffs for ADS (y-axis) and the percentage of predicted disordered residues (PPDR; x-axis). **B** Charge-Hydropathy and Cumulative Distribution Function (CH-CDF) analysis of AMPs and human proteome. The CH-CDF plot is a two-dimensional representation that integrates both the CH plot, which correlates a protein’s net charge and hydrophobicity with its structural order, and the CDF, which accumulates disorder predictions from the N-terminus to the C-terminus of a protein, offering insight into the distribution of disorder residues. The Y-axis (ΔCH) represents the protein’s distance from the CH boundary, indicating the balance between charge and hydrophobicity. It spans from -1.0 to 1.0, with positive values indicating disorder-promoting characteristics and negative values indicating order-promoting characteristics. The X-axis (ΔCDF) represents the deviation of a protein’s disorder frequency from the CDF boundary from -0.6 to 0.4, with positive values indicating a higher proportion of predicted disorder residues and negative values indicating a lower proportion of predicted disordered residues. Proteins are then stratified into four quadrants: Quadrant 1 (bottom right) indicates proteins likely to be structured; Quadrant 2 (bottom left) includes proteins that may be in a molten globule state or lack a unique 3D structure; Quadrant 3 (top left) consists of proteins predicted to be highly disordered; Quadrant 4 (top right) captures proteins that present a mixed prediction of being disordered according to CH but ordered according to CDF

To further analyze the propensity for intrinsic protein disorder in the AMPs and human proteome, we utilized PONDR® VSL2 percent and scores (Fig. 3). In the AMPs, 885 (64.46%) proteins were classified as highly disordered, with an average disorder score (ADS) > 0.5 or percent of disordered residues (PPDR) > 30%. Similarly, 12,386 proteins (60.86%) in the human proteome were categorized as highly disordered. A smaller subset of proteins—50 (3.64%) in the AMPs and 1,029 (5.06%) in the human proteome—were identified as moderately ordered or slightly flexible, indicating a lesser segment of each proteome with this characteristic.

Statistical analysis of these quadrant distributions using the χ^2^ test showed significant variations in how proteins were categorized between the AMPs and human proteome (χ^2^ value of 11.98, P-value 0.0025, with 2 degrees of freedom (DoF)). The DoF was calculated as (r-1) × (c-1), where *r* represents rows (the two groups: the AMPs and the human proteome) and *c* represents columns (the three distinct classifications: highly ordered, moderately disordered, or highly disordered). Thus, the final DoF value was 2.

#### Analysis of Context-dependent Binding Behavior of Proteins (FuzPred)

Next, we used FuzPred to further analyze the functional differences between the human proteome and the AMP dataset. The output metrics for this web page include pDO median and pDD median for each protein. pDO (probability of disorder-to-order transition) and pDD (probability of disorder-to-disorder transition). The pDO value indicates the likelihood that a disordered protein region will adopt a well-defined structure upon binding, while the pDD value represents the probability that a disordered region will remain heterogeneous or dynamic in the bound state. A Mann–Whitney U test was run for pDO and pDD comparing average medians of the AMPs to the human proteome (Table 2).

**Table 2:** Comparison of average redox and protein disorder values between AMP and human datasets using AIUPred Redox and FuzDrop/FuzPred, with statistical significance assessed using the Mann–Whitney *U* test

Category	AMP average	Human average	p-value
Redox-minus	0.328	0.396	< 0.0001*
Redox-plus	0.468	0.497	< 0.0001*
Redox-∆	− 0.140	− 0.101	< 0.0001*
pLLPS	0.316	0.489	< 0.0001*
pDO median	0.692	0.704	0.560
pDD median	0.314	0.298	0.224

*Statistically significant, p-value < 0.05

Significant differences were found for redox-minus, redox-plus, redox-∆ and pLLPS values, while pDO median and pDD median values did not show significant differences

Redox-∆: (Redox-∆ = redox-minus—redox-plus)

While our analysis examined a total of 20,435 human proteins and 3167 AMPs, only 20,274 and 410 sequences, respectively, contained the proper number of values to be run through FuzPred. The remaining proteins were filtered out due to size. The average pDO median for the AMPs was 0.692. The Human Average of 0.704, Mann–Whitney *U* Statistic of 4,086,327.0, and a p-value of 0.560. The pDD median produced an AMP Average of 0.314, Human Average of 0.298, Mann–Whitney *U* Statistic of 4,301,603.0, and a p-value of 0.224. Each of these metrics was found to be statistically insignificant.

#### Analysis of Protein LLPS Probability (FuzDrop)

FuzDrop was also used to assess the functional differences between the human proteome and AMPs. FuzDrop can predict the likelihood for a protein to undergo LLPS by sequence-based identification of droplet-promoting regions and aggregation-promoting regions within droplets.

As with FuzPred, FuzDrop required a certain length to properly examine and compare the provided sequences. Since these two tools are similar in their function, the same amount of protein sequences were able to be read by FuzDrop. 20,274 proteins were assessed for the human proteome, and 410 proteins were assessed for the AMP dataset. The main metric examined for this web page was the propensity of a protein to undergo LLPS. The pLLPS produced an AMP Average of 0.316, Human Average of 0.489, Mann–Whitney *U* Statistic of 2,715,042.0, and a p-value 2.21 × 10^–33^. This analysis was determined to be statistically significant based on the Mann–Whitney *U* Statistic and resulting p-value (Table 2).

#### Analysis of Redox Reactions with AIUPred

The redox tool of AIUPred was used to further assess and compare the human proteome to the AMP database. While 20,435 human proteins were initially analyzed, only 20,424 proteins contain the proper length for AIUPred processing. The AMP dataset began with 3167 proteins and was reduced to 3069 proteins following filtration based on size. This tool produced three metrics that were further examined by the Mann–Whitney *U* test. The redox-minus values indicate how disordered the proteins are under oxidizing conditions. The redox-plus values indicated how disordered the proteins are under reducing conditions. Finally, the redox-∆ is the difference between these two values for a given protein region. It quantifies how much a protein’s disorder changes between oxidizing and reducing environments. A higher absolute value of redox-∆ indicates that the protein region is highly sensitive to the redox state, meaning it undergoes significant structural changes depending on the oxidative conditions.

Table 2 presents a comparison of average redox values between AMP and Human datasets using AIUPred Redox, with statistical significance assessed using the Mann–Whitney *U* test. Significant differences were observed in both redox-minus (AMP: 0.328, Human: 0.396; p < 0.0001*) and redox-plus (AMP: 0.468, Human: 0.497; p < 0.0001*), indicating distinct oxidative potential profiles between the two groups. These findings highlight notable disparities in redox properties, underscoring potential functional differences that may influence cellular processes and disease mechanisms. The lower redox-minus values in AMPs suggest they may be more resistant to reduction, potentially enhancing their stability and functionality in reducing environments, which could be crucial during infection or inflammation. Conversely, the higher redox-plus values in humans suggest a greater vulnerability or responsiveness to oxidative conditions, which could impact cellular signaling pathways and responses to oxidative stress.

Table 2 further explores the comparison by focusing on average redox-∆ values between AMP and Human datasets, calculated as the difference between redox-minus and redox-plus. The Mann–Whitney *U* test revealed a statistically significant difference (AMP: − 0.140, Human: − 0.101; p < 0.0001*), indicating a more pronounced redox imbalance in the AMPs compared to the Human proteome. This differential redox state could signify adaptive responses or regulatory mechanisms specific to AMPs as compared to the human proteome. A greater redox-∆ in AMPs suggests they have adapted to more rapidly switch between reduced and oxidized states, which may confer advantages in dynamic microbial environments where rapid responses to oxidative stress are necessary. This flexibility could facilitate AMPs’ roles in host defense by enabling rapid activation or deactivation of their antimicrobial functions depending on the redox status of their environment.

### CH-CDF Plot Analysis

Using the CH-CDF analysis to categorize the AMPs and human proteome, a total of 21,725 proteins were distributed into four quadrants according to their predicted disorder characteristics. The y-axis (ΔCH) spans from − 1.0 to 1.0, representing the balance between net charge and hydrophobicity. Positive values indicate disorder-promoting characteristics, while negative values suggest order-promoting characteristics. The x-axis (ΔCDF) spans from − 0.6 to 0.4, reflecting the deviation from the CDF boundary. Positive values indicate a higher proportion of predicted disordered residues, while negative values suggest a lower proportion of disordered residues.

The AMP analysis showed that 772 proteins (56.23%) were assigned to Quadrant 1 (Bottom Right), indicating a structured state. Quadrant 2 (Bottom Left), containing 121 proteins (8.81%), corresponded to proteins in a molten globule state or lacking a distinct 3D structure. Quadrant 3 (Top Left) included 232 proteins (16.9%) associated with high disorder or lack of structure, while Quadrant 4 (Top Right) had 248 proteins (18.06%) exhibiting a mix of ordered and disordered predictions.

For the human proteome, 12,030 proteins (59.11%) were in Quadrant 1 (Bottom Right), indicating a structured state. Quadrant 2 (Bottom Left) contained 5,188 proteins (25.49%) characterized as molten globules, and Quadrant 3 (Top Left), with 2,508 proteins (12.32%), signified a high level of disorder. Finally, Quadrant 4 (Top Right) had 626 proteins (3.08%) with mixed structural characteristics.

A χ^2^ test was conducted to evaluate the distribution of proteins across the different structural classes in the CH-CDF plot. The results, with a χ^2^ value of 887.66 and a P-value < 0.001 for 3 degrees of freedom, demonstrated a significant discrepancy from the expected distribution in the quadrants. The degrees of freedom were calculated using the formula DoF = (r−1) × (c−1), with *r* denoting rows and *c* denoting columns in a table. The two rows once again represent the two different groups: the AMPs and the human proteome. The columns represent the four distinct quadrants: Q1, Q2, Q3, and Q4. The product of the differences between these values gives DoF of 3. This significant difference indicates that the protein distribution pattern across the quadrants is not due to random variation, suggesting distinctive differences in the structural states of the proteins within the AMPs and human proteome when compared to what would occur by chance. Moreover, the marked structural variance between the AMP and human proteomes highlighted by this analysis could influence how these proteins respond to environmental stresses and pathogenic challenges, potentially impacting their evolutionary adaptations and roles in disease processes.

### Length-Adjusted Comparison of Human and AMP Proteomes

A subset of 3,094 human proteins, with sequence lengths ranging from 31 to 183 amino acids, was selected to match the range observed in AMP. Statistical analysis revealed significant differences across all RIDAO metrics, with AMPs and the length-adjusted human proteome showing distinct patterns in disorder and phase separation metrics (P < 0.001 for all comparisons).

The CH-CDF plot categorized proteins based on structural tendencies using a Cartesian coordinate system. Proteins in Quadrant 1 (Q1, bottom right) are structured, with negative CH scores and positive CDF scores. Quadrant 2 (Q2, bottom left) includes molten globules or hybrids, characterized by negative scores for both CH and CDF. Quadrant 3 (Q3, top left) represents highly disordered proteins with positive CH scores and negative CDF values, while Quadrant 4 (Q4, top right) includes proteins with conflicting indicators, classified as disordered in CH but ordered in CDF.

Chi-squared analysis of quadrant classifications revealed significant differences in protein distribution (χ^2^ = 378.86, P < 0.001, degrees of freedom = 3). AMPs were predominantly categorized in Q1 (56.23%) and Q3 (16.90%), reflecting a higher proportion of structured and highly disordered proteins. In contrast, the matched subset showed higher proportions in Q2 (23.82%), associated with molten globules, and Q1 (54.07%). Notably, 18.06% of AMPs were classified in Q4, compared to only 3.30% of the matched subset.

Structural classification further highlighted differences between the two groups. Among AMPs, 885 proteins were classified as “Highly Disordered,” compared to 2,080 proteins in the matched subset. None of the AMPs were categorized as “Highly Ordered,” whereas 14 proteins in the matched subset fell into this classification. AMPs also had fewer proteins in the “Moderately Ordered or Mildly Flexible” category (50 for AMPs vs. 143 for the matched subset).

When controlling for sequence length and comparing LLPS and binding propensities, AMPs exhibited significantly lower values for all metrics compared to the human subset. The mean pLLPS for AMPs was 0.10 (IQR: 0.00–0.13), while for the length-matched human proteins, it was 0.39 (IQR: 0.14–0.63). For pDO_median, the mean for AMPs was 0.23 (IQR: 0.00–0.64), compared to 0.68 (IQR: 0.59–0.83) for human proteins. Similarly, the mean pDD_median for AMPs was 0.10 (IQR: 0.00–0.18), whereas for human proteins, it was 0.32 (IQR: 0.18–0.41). The Mann–Whitney U test revealed statistically significant differences between AMPs and the length-matched human proteome subset for all metrics (p < 0.0001), confirming that these differences persist even when controlling for sequence length.

## Discussion

Our findings shed new light on AMPs, shifting our understanding of them as solely antimicrobial agents to dynamic, multifunctional proteins that share critical features with intrinsically disordered proteins. This study demonstrates that AMPs exhibit a significant degree of intrinsic protein disorder and propensity for LLPS, characteristics that enable rapid structural adaptability in response to environmental cues, such as oxidative stress. These properties suggest that AMPs have functional versatility, which may be deeply tied to their disordered nature. These features may allow AMPs to target pathogens effectively and to adapt their structure to enhance immune modulation and pathogen interaction. By characterizing the inherent flexibility of the AMPs, our work reshapes the understanding of how AMPs operate at a molecular level as they function in the immune response with roles that go beyond conventional antimicrobials.

Expanding beyond the conventional focus of proteomic studies, our study offers a layer of analysis that surpasses protein identification and quantification. Through the evaluation of a large set of proteins, we introduced a novel conceptual framework for comparing the disorder profiles of antimicrobial peptides and the human proteome. Our study revealed distinct amino acid composition profiles and significant differences in the intrinsic disorder profiles between the two groups analyzed.

Although there was an enrichment of cysteine (C) levels in both the AMP and human proteomes, there was a significantly higher representation of cysteine in the AMPs. This may contribute to the preservation of the structural integrity of these proteins while allowing for the anatomical changes necessary for immune modulation and antimicrobial activity [49, 50]. There was also greater enrichment of arginine (R) in the AMPs relative to the human proteome. A previous study found that even tripeptides consisting of two arginine and one glycine exhibit antimicrobial activity, and poly-L-arginine, a synthetic polymer made of multiple units of arginine, has potent activity against both *E. coli* and *S. aureus* [51, 52]. The heavier enrichment of arginine found in the AMPs may suggest enhancement of antimicrobial characteristics when compared to the human proteome. The richness of proline, a disorder-promoting residue, in the human proteome suggests a propensity for structural disorder, a greater degree of elasticity, and an enrichment in beta turns [53, 54]. This characteristic may be attributed to the dynamic functions of the human proteome, which are essential for maintaining homeostasis within the body. Furthermore, dynamic changes in structure are necessary for the human proteome to adapt to various physiological conditions, interact with a wide range of molecular partners, and perform diverse biochemical functions effectively. These differences in amino acid composition profiles between the AMPs and the human proteome reflect the distinct functional requirements of each group.

Another key finding of our analysis included AMP’s higher propensity for intrinsic protein disorder compared to the human proteome. This underscores a unique aspect of their functional properties. Previous research has suggested that intrinsic disorder plays an important role in proteins with antimicrobial properties. For example, caseins, a group of proteins known to be intrinsically disordered, are also known to exhibit antimicrobial properties [55, 56]. Another study found that NK-lysins, which are known AMPs, had significantly elevated levels of intrinsic disorder [57]. Yet another study focusing on the ability of human skin to resist common bacteria found that any isolated linear cationic peptides on the skin surface that contained IDRs also had potent anti-microbial activity [51]. The positive charges in these AMPs enhance their electrostatic affinity and facilitate their binding, while their hydrophobicity and overall amphiphilicity help them integrate into and disrupt the lipid bilayers of bacterial targets [58]. These properties underline why antimicrobial peptides, with their broad-spectrum antibacterial activity, offer a promising future in overcoming antibiotic resistance and are less prone to drug resistance [58, 59]. The dynamic and structurally diverse nature of AMPs, paired with their ability to adapt various conformations to enhance their interaction with bacterial cell membranes, almost necessitates a degree of intrinsic disorder. It is also plausible that IDRs within AMPs may play significant roles in signal transduction pathways used to modulate the host immune response. Due to the clear overlap in function between these IDPs and AMPs, it is not surprising to see a significant degree of intrinsic disorder within the AMPs.

AMPs and human innate immune proteins such as defensins and histatins are pivotal in innate immunity, leveraging their unique structural features to disrupt microbial cell integrity. AMPs often adopt amphipathic helices that interact with and destabilize microbial membranes through pore formation or membrane disruption [59]. In contrast, defensins feature a beta-sheet-rich structure stabilized by disulfide bonds, which facilitates their insertion into microbial membranes to form pore-like structures, leading to cell lysis [60]. Histatins, primarily active against fungi, rely on their ability to bind to fungal cell walls and subsequently penetrate to disrupt mitochondrial energy production [61]. These molecular mechanisms highlight how the structural diversity of these peptides underpins their antimicrobial strategies, offering pathways for therapeutic innovation against a broad spectrum of pathogens.

The PONDR® and CH-CDF analyses reveal a broad spectrum of protein structures within the antimicrobial peptides and human proteome, highlighting their structural diversity from highly structured to various degrees of disorder, including adaptable molten globules. This variety suggests a dynamic interplay essential for their biological functions. AMPs are crucial in innate immunity, targeting pathogens, whereas human proteins support diverse physiological processes like cellular regulation and signaling. Both groups exhibit intrinsic disorder, facilitating complex interactions within cellular networks and enabling proteins to engage in multiple regulatory and signaling pathways. The prevalence of intrinsic disorder in the human proteome is significant, and such proteins are often implicated in critical cellular functions. Moreover, their flexible nature is associated with various pathologies. Dysregulation or mutation of IDPs can lead to diseases such as cancer, cardiovascular diseases, neurodegenerative disorders like Alzheimer’s and Parkinson’s diseases, and diabetes [62–65].

Our analysis shows a significant difference in redox-∆ values between AMP and human proteins, indicating distinct responses to oxidative and reductive environments. The negative redox-∆ for AMPs suggests they become less disordered under oxidizing conditions, contributing to their structural stability in oxidative environments, such as when combating pathogens. This stability may enhance AMP functionality in oxidative settings like inflamed tissues or immune responses to infection, where reactive oxygen species (ROS) are present and are commonly released during an attack on a pathogen by the host immune system [66–68]. AMP’s heightened sensitivity to redox changes might also suggest an increased ability to function in varied niches, such as mucosal surfaces, the gut, or areas of inflammation, where redox conditions can shift dramatically [69]. Conversely, human proteins have a smaller redox-∆ values, indicating less variability in intrinsic disorder between oxidized and reduced states despite being slightly more disordered when existing in either state. This may protect critical cellular processes such as metabolic reactions and signal transduction from being overly disrupted by oxidative shifts and fluctuating redox environments, while maintaining levels of intrinsic disorder that may be necessary to perform these tasks [70, 71]. Furthermore, for human proteins, stability across redox states suggests an evolutionary preference for maintaining critical cellular functions without risking conformational shifts that could interfere with essential processes like energy production or DNA repair [72].

In addition to redox-∆ variations between AMPs and human proteins, the propensities for their respective proteins to undergo LLPS was found to differ. Greater disorder in a protein can be associated with a higher pLLPS [73–75]. The amino acid sequence and availability of multiple binding interactions can determine how likely a protein is to undergo LLPS [74, 76, 77]. This process allows for compartmentalization of reactions which occur in cellular locations not bound by membrane, thus subcellular reactions can be concentrated and catalyzed based on its partners and environment [74, 76, 77]. Our analysis showed that the propensity for LLPS is higher in human proteins compared to AMPs. This may suggest that human proteins are more predisposed to forming membrane-less organelles, such in stress granules or signaling complexes [78, 79]. In contrast, while AMPs show a lower overall propensity for LLPS, they still require structural flexibility to form dynamic organelles in response to environmental stimuli. However, this ability to phase separate may be more controlled and directed towards areas of concentrated antimicrobial activity within specific cellular regions [80, 81]. Ultimately, formation of these transient compartments is still necessary for antimicrobial function in pathogen-rich environments [82]. These findings may suggest that a higher propensity for LLPS in human proteins may enable widespread organelle formation across cells, as opposed to directed antimicrobial activity for AMPs.

The results from the length-adjusted analysis reaffirm the distinct biophysical and structural characteristics of AMP compared to human proteins. By controlling for sequence length, we eliminated a key confounding factor, further solidifying the conclusion that AMPs possess significantly higher levels of intrinsic disorder and unique LLPS propensities. The CH-CDF plot analysis highlighted that AMPs occupy structural niches distinct from human proteins, with a notable enrichment in Quadrant 3, representing highly disordered proteins, and Quadrant 4, reflecting a combination of disordered and ordered characteristics. This distribution underscores the functional versatility of AMPs, which may leverage their structural adaptability to interact with diverse microbial targets under varying environmental conditions.

Statistically significant differences in LLPS propensity metrics (P < 0.0001), such as lower pLLPS and binding transition probabilities (pDO and pDD) in AMPs, suggest a reduced but more targeted phase separation capability. This aligns with the hypothesis that AMPs are evolutionarily optimized for specific, localized functions, such as forming biomolecular condensates in pathogen-rich environments to enhance antimicrobial efficacy. Conversely, the higher LLPS propensities observed in the human proteome may reflect broader roles in cellular compartmentalization and signaling, indicative of their diverse physiological functions. These findings suggest that intrinsic disorder and LLPS propensity serve as defining features of AMPs, enabling their rapid adaptability and potent antimicrobial activity in dynamic environments.

This length-controlled analysis provides compelling evidence that intrinsic disorder and LLPS propensity are central to the functional divergence between AMPs and human proteins, independent of sequence length. These findings not only enhance our understanding of AMP functionality but also pave the way for future studies to explore the therapeutic potential of engineered peptides that mimic the biophysical properties of AMPs. Further investigations could examine the impact of other sequence features, such as charge distribution and hydrophobicity, to refine our understanding of the structure–function relationship in AMPs.

## Limitations

While our analysis provides useful insights into the structural and functional diversity of both AMPs and the human proteome, it is not without limitations. One limitation of our study is that there may be variation with the disorder predictor software that was utilized. Disorder predictor models are developed with both training and testing data sets, which are not standardized across predictors. Performances among different disorder predictors may vary, particularly when different data sets are used to train the predictors. However, we aimed to mitigate this potential variability in two ways: by utilizing multiple disorder predictors and by calculating the mean disorder profile for both ADS and PPDR. These served as aggregate scores for all the utilized disorder predictors, intended to help control for undesired variations in predictor output.

Our analyses are also heavily dependent on the accuracy and completeness of both the online databases and the datasets used by our tools, such as PONDR® VSL2B and the Composition Profiler. Any errors or biases in these sources may affect our results and subsequent interpretations of the data. Additionally, the predictive nature of bioinformatics tools means that our findings are based on computational models rather than experimental validation. Although our disorder predictors provide robust predictions, it is impossible for them to fully capture the complexity of protein behavior in vivo. The dynamic environment of different tissues may influence protein behavior differently. Finally, our use of static datasets does not account for temporal changes in protein expression, post-translational modifications, or interactions that could influence the functional properties of proteins. Consequently, experimental studies are needed to truly observe levels of intrinsic disorder and the ultimate behavior of these proteins in physiologic environments. Future directions should focus on identifying specific AMPs and evaluating them for intrinsic disorder, as well as development of standardized methodologies for bioinformatic analysis. We hope that deeper understanding of the behavior of these proteins can eventually lead to targeted therapeutic drug development.

## Conclusion

Our study provides a detailed comparative analysis of intrinsic disorder and LLPS propensity in AMPs and human proteins, uncovering the unique structural adaptations that distinguish these two. While both exhibit significant levels of intrinsic disorder, which likely play crucial roles in their biological functions, we found that AMPs demonstrate a markedly higher degree of flexibility. This appears to be essential to their rapid and effective antimicrobial functions as the heightened disorder enables AMPs to adopt multiple conformations, facilitating their interactions with various microbial targets and allowing them to operate efficiently in diverse and often hostile environments, such as those with high oxidative stress or complex microbial communities.

## Data Availability

No datasets were generated or analysed during the current study.

## Abbreviations

VRE: Vancomycin-resistant enterococcus
MRSA: Methicillin-resistant *Staphylococcus aureus*
AMPs: Antimicrobial peptides
IDPs: Intrinsically disordered proteins
IDRs: Intrinsically disordered regions
LLPS: Liquid–liquid phase separation
UniProt: Universal protein resource
CP: Composition profiler
RIDAO: Rapid intrinsic disorder analysis online
ADS: Average disorder scores
PPDR: Percentages of predicted disordered residues
PONDR®: Predictor of natural disordered region
FZP: FuzPred
pDO: Disorder-to-order
pDD: Disorder-to-disorder
MBM: Multiplicity of binding modes
pLLPS: Liquid–liquid phase separation propensity
DPRs: Droplet-promoting regions
CH: Charge-hydropathy
CDF: Cumulative distribution function
SP: Swiss-prot
ANOVA: Analysis of variance
AIUPred: Artificial intelligence-based predictor of intrinsic disorder
CASP: Critical assessment of protein structure prediction
AAC: Amino acid composition
IDRs: Intrinsically disordered regions
PDB: Protein data bank
PTMs: Post-translational modifications
Pfam: Protein families
MWU: Mann–Whitney U
DoF: Degrees of freedom
